# Kinetic Limits of Graphite Anode for Fast-Charging Lithium-Ion Batteries

**DOI:** 10.1007/s40820-023-01183-6

**Published:** 2023-09-22

**Authors:** Suting Weng, Gaojing Yang, Simeng Zhang, Xiaozhi Liu, Xiao Zhang, Zepeng Liu, Mengyan Cao, Mehmet Nurullah Ateş, Yejing Li, Liquan Chen, Zhaoxiang Wang, Xuefeng Wang

**Affiliations:** 1 Beijing National Laboratory for Condensed Matter Physics, Institute of Physics, Chinese Academy of Sciences, Beijing, 100190 People’s Republic of China; 2 School of Physical Sciences, University of Chinese Academy of Sciences, Beijing, 100049 People’s Republic of China; 3 College of Materials Science and Opto-Electronic Technology, University of Chinese Academy of Sciences, Beijing, 100049 People’s Republic of China; 4 Department of Chemistry, Boğazici University, Bebek, Istanbul, 34342 Türkiye; 5 Tianmu Lake Institute of Advanced Energy Storage Technologies Co. Ltd., Liyang, 213300 People’s Republic of China

**Keywords:** Fast-charging, Graphite anode, Cryogenic transmission electron microscopy (cryo-TEM), High-rate kinetics

## Abstract

Fast-charging lithium-ion batteries are highly required, especially in reducing the mileage anxiety of the widespread electric vehicles. One of the biggest bottlenecks lies in the sluggish kinetics of the Li^+^ intercalation into the graphite anode; slow intercalation will lead to lithium metal plating, severe side reactions, and safety concerns. The premise to solve these problems is to fully understand the reaction pathways and rate-determining steps of graphite during fast Li^+^ intercalation. Herein, we compare the Li^+^ diffusion through the graphite particle, interface, and electrode, uncover the structure of the lithiated graphite at high current densities, and correlate them with the reaction kinetics and electrochemical performances. It is found that the rate-determining steps are highly dependent on the particle size, interphase property, and electrode configuration. Insufficient Li^+^ diffusion leads to high polarization, incomplete intercalation, and the coexistence of several staging structures. Interfacial Li^+^ diffusion and electrode transportation are the main rate-determining steps if the particle size is less than 10 μm. The former is highly dependent on the electrolyte chemistry and can be enhanced by constructing a fluorinated interphase. Our findings enrich the understanding of the graphite structural evolution during rapid Li^+^ intercalation, decipher the bottleneck for the sluggish reaction kinetics, and provide strategic guidelines to boost the fast-charging performance of graphite anode.

**Supplementary Information:**

The online version contains supplementary material available at 10.1007/s40820-023-01183-6.

## Highlights

- The microstructure of graphite upon rapid Li^+^ intercalation is a mixture of differently staging structures in the macroscopic and microscopic scales due to the incomplete and inhomogeneous intercalation reactions hindered by the sluggish reaction kinetics.
- The Li^+^ interface diffusion dominates the reaction kinetics at high rates in thin graphite electrode, while Li^+^ diffusion through the electrode cannot to be neglected for thick graphite electrode.

**Supplementary Information:**

The online version contains supplementary material available at 10.1007/s40820-023-01183-6.

## Graphical abstract

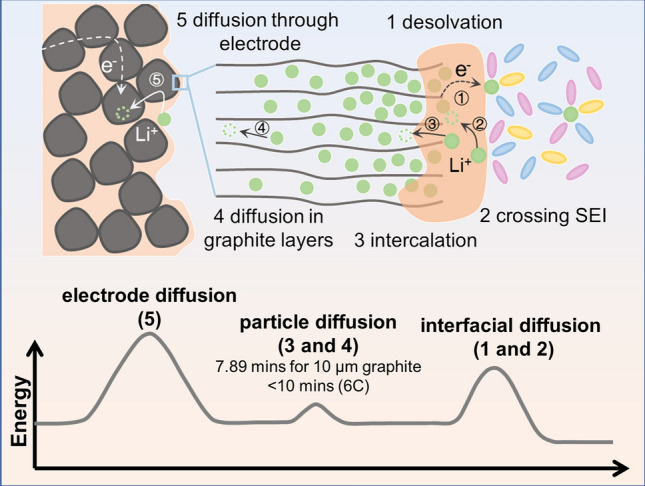

## Introduction

Charging lithium-ion batteries (LIBs) in a fast and safe manner is critical for the widespread utility of the electric vehicles [1–5]. However, fast Li^+^ intercalation in graphite is challenging due to its sluggish kinetics [6–8]. When charged at high rates, the graphite anode suffers from large polarizations, low intercalation capacity, and deteriorating side reactions including lithium metal plating, severe solid electrolyte interphase (SEI) formation, and Joule heating [9–11]. It is essential to accelerate the Li^+^ intercalation kinetics and reduce its polarization in order to avoid these problems and achieve a high-performance graphite anode for fast-charging LIBs.

**Fig. 1:**
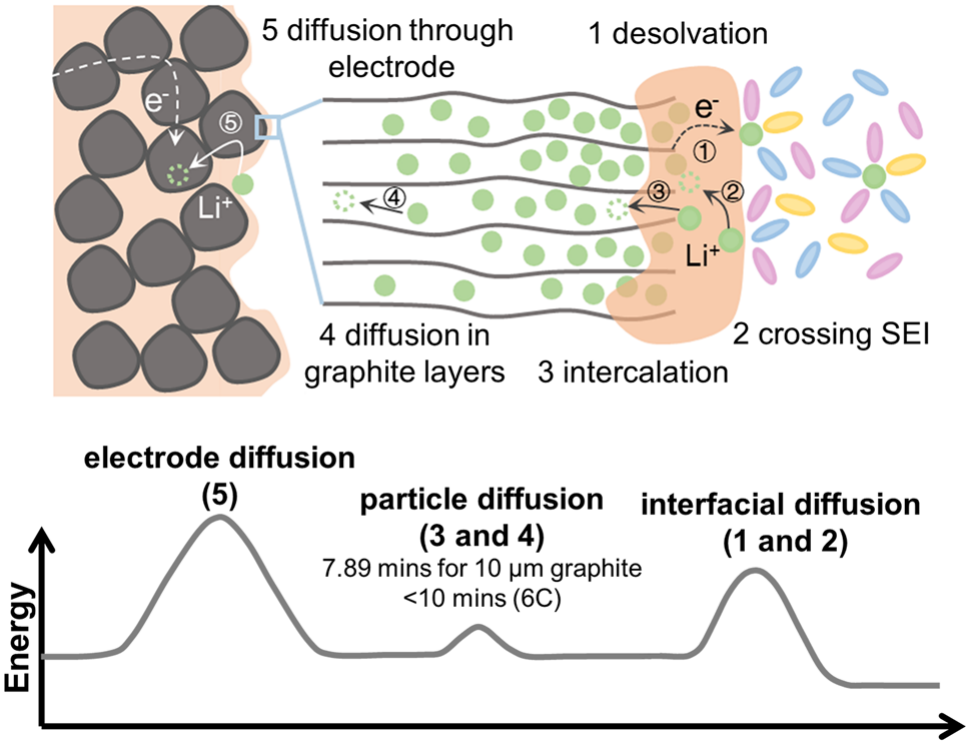
Schematic illustration of the Li^+^ intercalation in graphite, including Li^+^ interfacial diffusion, particle diffusion, electrode diffusion, and their corresponding energy consumption

During Li^+^ intercalating into graphite, the Li^+^ experiences several steps, including desolvation, crossing the SEI, intercalation and diffusion in graphitic layers, and transport through the electrode (Fig. 1) [3, 9]. These processes can be further categorized into three main energy-consuming steps: interfacial diffusion, particle diffusion, and electrode diffusion. Some of them occur concurrently and are difficult to deconvolute, such as Li^+^ desolvation and crossing the SEI due to the porous nature of SEI layer [9, 12–14]. The interfacial diffusion is regulated by the electrolyte chemistry and SEI property [15–18], while the particle diffusion is closely related to the Li^+^ diffusion into graphene layers as well as the transition of different staging structures [19–21]. A recent work revealed the localized-domain nature of the staged graphite and implied that fast Li^+^ intercalation into graphite particle is intrinsically possible [22]. However, the structural evolution of graphite upon fast Li^+^ intercalation is unclear, while the main rate-determining step is still in controversy, both of which are fundamental for improving the reaction kinetics of graphite [14, 15].

In this sense, we decoupled the forementioned three Li^+^ diffusion and uncovered the structural/interfacial evolution and reaction limit of graphite at high current densities by cryogenic transmission electron microscopy (cryo-TEM) and other techniques. The results show that fast Li^+^ intercalation leads to incomplete and inhomogeneous intercalation reactions and the Li^+^ is mainly localized in the subsurface of the graphene layers as evidenced by electron energy loss spectroscopy (EELS) mapping. The Li^+^ diffusion coefficient through the particle was determined and found it quick enough to support the fast intercalation at least 6 C when the particle size is less than 10 μm. Consequently, Li^+^ diffusion across the interface and electrode becomes the main rate-determining steps; the former is highly dependent on the electrolyte chemistry and can be enhanced by the LiF-rich SEI layer, while the latter is related to the electrode compactness. Our findings renew the understanding of the microstructure of graphite upon rapid Li^+^ intercalation and the reaction bottlenecks and shed light on boosting the fast-charging capability of graphite by constructing a fluorinated interphase and electrode engineering.

## Experimental Section

### Material Preparation Process

The working electrode sheets were prepared by mixing the KS6 graphite powder (TIMCAL Graphite & Carbon) and polyvinylidene fluoride (PVDF) dissolved in N-methyl pyrrolidone (NMP) at a weight ratio of 9:1 and then casting the slurry onto a piece of copper foil. The foil was vacuum-dried at 120 °C for 6 h. The electrodes were punched with a diameter of 10 mm, a thickness of 68 μm, and active material areal loading of ~ 2.5 mg cm^−2^. The graphite electrodes for the *in situ* XRD test were prepared by mixing KS6 graphite powder and polytetrafluoroethylene (PTFE) binder at a mass ratio of 95:5. The composites were ground into square thin slices with a width of 10 mm and an active material loading of ~ 10 mg cm^−2^ and then dried at 120 °C under vacuum for 6 h.

Battery-grade lithium hexafluorophosphate (LiPF_6_), ethylene carbonate (EC), dimethyl carbonate (DMC), and fluoroethylene carbonate (FEC) were ordered from DodoChem Technology. These reagents were used as received without purification. LiPF_6_–EC/DMC electrolyte was prepared by dissolving 1.0 mol L^−1^ LiPF_6_ in EC/DMC (1:1, *v*/*v*) in an argon-filled glove box (MBraun Lab Master 130; the contents of O_2_ and H_2_O were both lower than 0.1 ppm). FEC (10 vol%) was added into LiPF_6_–EC/DMC electrolyte to prepare LiPF_6_–EC/DMC + FEC electrolyte.

### Electrochemical Evaluation

Coin cells (CR2032) were assembled with KS6 graphite as the working electrode, fresh Li foil as the counter electrode, a polypropylene film (Celgard 2400) as the separator, and LiPF_6_–EC/DMC or LiPF_6_–EC/DMC + FEC as the electrolyte in an Ar-filled glove box.

The electrochemical cycling was performed on a Neware battery test system (CT-4008 T-5V10mA-164 and CT-4008 T-5V50mA-164, Shenzhen, China) in the galvanostatic mode between 0.0 and 3.0 V (*vs.* Li^+^/Li^0^). The cycled graphite electrodes were taken out from the cells, rinsed with DMC, and dried in the vacuum mini-chamber of the glove box before the postmortem characterization.

The electrochemical impedance spectra (EIS) and potential relaxation technique (PRT) were conducted on an electrochemical workstation (BioLogic SP-200 system, France) using a three-electrode setup (graphite electrode as the working electrode and fresh Li foil as the counter and reference electrodes) at 20 °C. Before the potential relaxation measurement, the cells were pre-cycled and then discharged/charged to a certain potential at 0.05 C. After removing the current, the change of the open-circuit voltage (OCV, φ) over time (t) within 48 h was recorded. The initial potential change within 2 mV in 1 h was set as the equilibrium potential (the potential of the electrode as the relaxation time tends to infinity, φ_∞_). The voltage increases/decreases gradually and then stabilizes. The rapid voltage change is attributed to the Li^+^ diffusion in the electrolyte and short-range interface, while the subsequent slow change is ascribed to the Li^+^ diffusion through the long-range particle and electrode.

The chemical diffusion coefficient of Li^+^ in the particle bulk (*D*) can be calculated from the linear slope of $\ln \left[ {\exp \left( {\frac{{\varphi_{\infty } - \varphi }}{RT}F} \right) - 1} \right]$ over time as Eq. (1) [19]:

1 $$ \ln \left[ {\exp \left( {\frac{{\varphi_{\infty } - \varphi }}{RT}F} \right) - 1} \right] = - \frac{{\pi^{2} }}{{L^{2} }}Dt - \ln N $$

where *F* is the Faraday constant, *R* is the molar gas constant, *T* is the thermodynamic temperature, and *L* is the thickness of the electrode. $N = \frac{{\exp \left( { - \frac{{\pi^{2} }}{{L^{2} }}D\xi } \right)}}{{\exp \left( {\frac{{\varphi_{\infty } - \varphi_{\xi } }}{RT}F} \right) - 1}}$; when *t* = *ξ*, the voltage is ${{\varphi }}_{{\upxi }}$, which can be measured experimentally.

The time for Li^+^ diffusing from the surface to the bulk center of graphite with a diameter of *d* (*t*_diffusion_) is estimated by Eq. (2):

2 $$ t_{{{\text{diffusion}}}} = \frac{{(d/2)^{2} }}{{2D_{\min } }} $$

where *D*_min_ is the lowest Li^+^ diffusion coefficient measured in the experiment (Tables S2 and S3, *e.g., D*_min_ = 2.64 × 10^−10^ cm^2^ s^−1^ during Li^+^ intercalation process).

### Physical Characterization

The X-ray diffraction (XRD) was performed on the X’Pert-Pro MPD diffractometer with monochromatic Cu Kα radiation (λ = 1.541 Å). Cryogenic (scanning) transmission electron microscopy (cryo-(S)TEM) characterizations were carried out using a JEOL JEM-F200 microscope under cryogenic temperatures (− 180 °C) at 200 kV. The cycled graphite powder samples were scraped from the cycled graphite electrodes rinsed with DMC and loaded on the TEM grids. Then, the grid was transferred into the cryo-holder (Fischione 2550) in an Ar-filled glove box. Using a sealed container, the cryo-holder was quickly inserted into a JEOL JEM-F200 microscope. Liquid nitrogen was poured into the cryo-holder, and the sample temperature dropped and stabilized at about − 180 °C. The lattice spacings of graphite and other inorganic species were measured by Digital Micrograph (DM, Gatan) software. Inverse fast Fourier transform (iFFT) was performed to improve the signal-to-noise ratio. Strain analysis was performed based on the geometric phase analysis (GPA) method [23, 24] using the FRWR tools plugin (www.physics.hu-berlin.de/en/sem/software/software_frwrtools) in DM. ImageJ software was used to color the image except for the distortion region by contrast difference, and the defect fraction was obtained by counting the area ratio of the uncolored part to the whole area.

## Results and Discussion

### Structural Evolution of Graphite upon Fast Li^+^ Intercalation

**Fig. 2:**
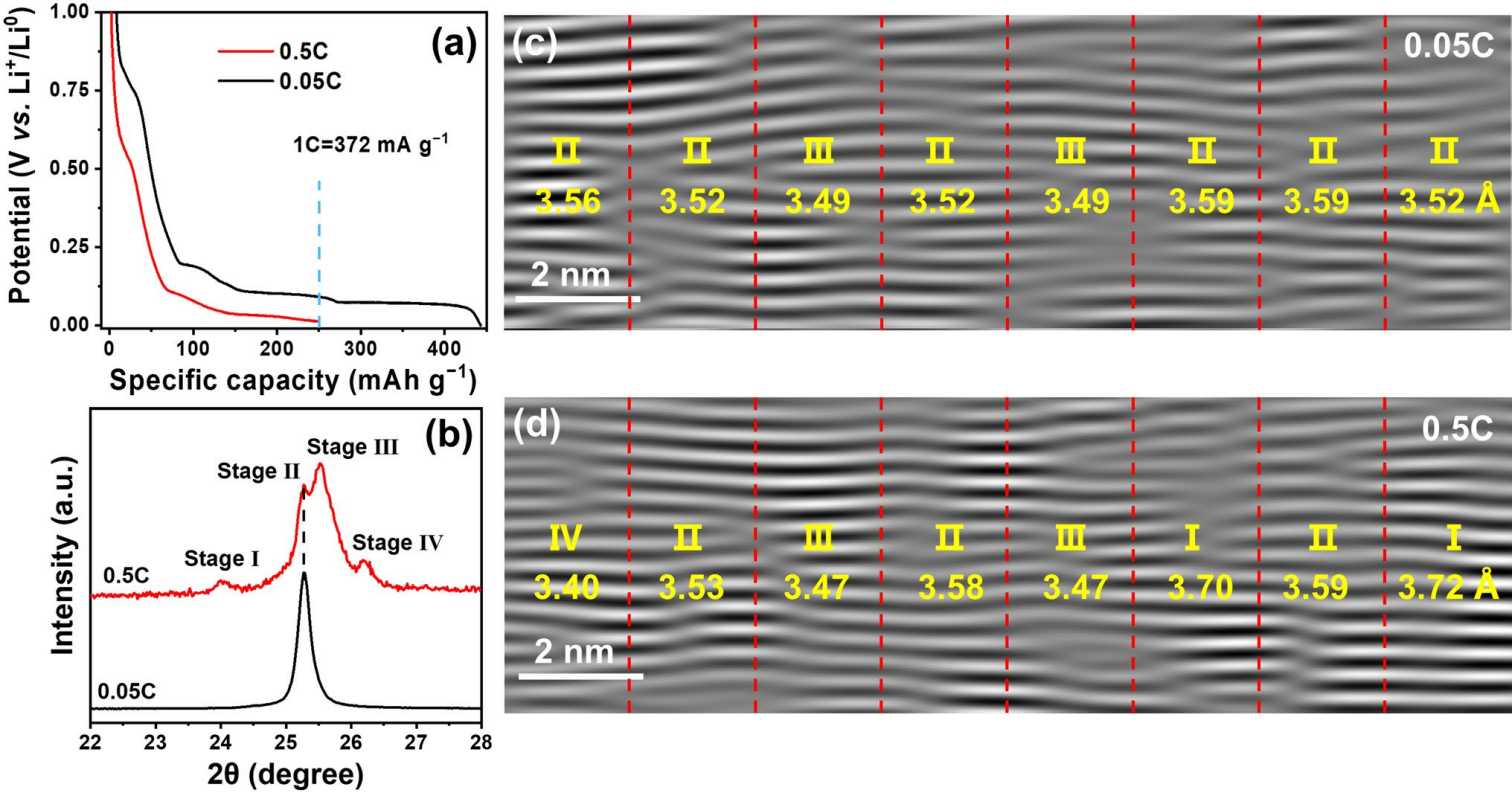
Structural comparison of graphite at low-rate (0.05 C) and high-rate (0.5 C) lithiation to the same capacity (250 mAh g^−1^). **a** The voltage curves, **b** XRD patterns, and **c**, **d** the iFFT images of the graphite discharged at 0.05 C (black) and 0.5 C (red). (Color figure online)

Commercial flaky graphite was used with an average particle size of 6 μm, and thin electrode with areal loading of ~ 2.5 mg cm^−2^ was prepared without rolling in order to compare the Li^+^ diffusion kinetics across the interface and particle while minimizing the diffusion length through the electrode. It was discharged at a low and high current density (0.05 C *vs.* 0.5 C, 1 C = 372 mA g^−1^, Fig. 2), respectively. Drastically increasing the Li^+^ intercalation rate by a magnitude leads to an increase in the cell polarization for about 70 mV, and thus, the intercalation reaction stops before the last plateau yielding a low initial capacity (Fig. 2a). The structures of the lithiated graphite were compared by XRD at the same lithiation content (250 mAh g^−1^). Coexistence of four staging phases is observed at 0.5 C, while single-phase Stage II is present at 0.05 C (Fig. 2b), indicating that the transition of different staging structures is limited by reaction kinetics, and rapid Li^+^ intercalation causes uneven Li^+^ distribution. This leads to the long-range structural heterogeneity of the lithiated graphite upon fast Li^+^ intercalation. The *in situ* XRD patterns at 0.5 C (Fig. S1) further confirm the coexistence and incomplete transition of different staging structures owing to the sluggish reaction kinetics.

Cryo-TEM characterizations proved the concomitance of different staging structures in the microstructure of the lithiated graphite, especially when discharged at 0.5 C (Figs. 2c–d and S2). Based on the average interplane spacings (based on 11 graphene layers), the corresponding specific staging structures were identified and assigned in different domains of the lithiated graphite (Fig. 2c, d). As shown in Fig. 2d, Stage I-to-IV structures are present concurrently in the graphite discharged at 0.5 C, exhibiting a significant heterogeneity at the nanoscale. Such heterogeneity is extended to different regions of a single particle and to different particles, reflected by the uneven distribution of defective structure and strains caused by the Li^+^ intercalation [22] (Fig. S2). In contrast, Stage II structure is dominant in the graphite discharged at 0.05 C (Fig. 2c), consistent with the XRD result (Fig. 2b).

**Fig. 3:**
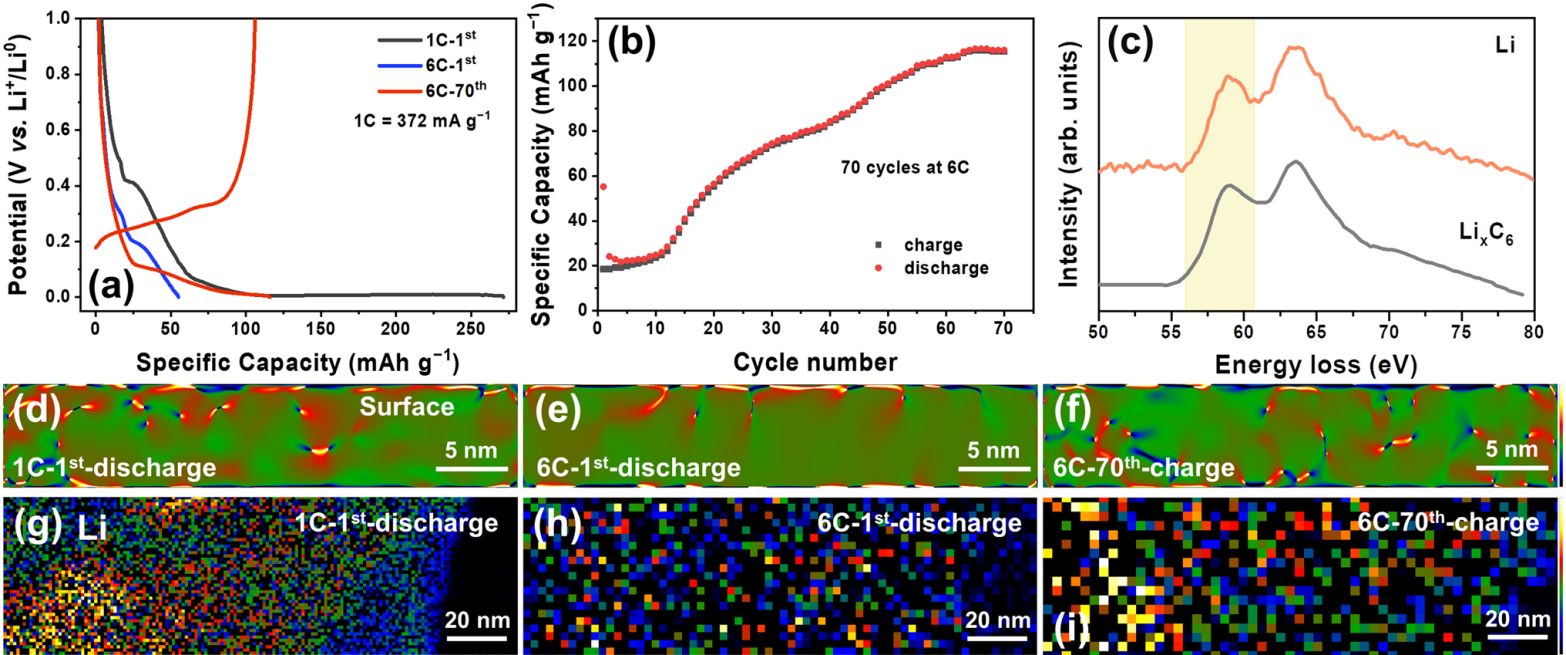
**a** Potential curves of the graphite discharged at 1 C and 6 C. **b** Cycling performance of graphite cycled at 6 C. **c** EELS spectra of Li. The spectrum of Li_x_C_6_ (gray) was obtained during slow lithiation (discharge to 0 V at 0.05 C), and the orange one was measured at 1 C. The characteristic peak at around 58 eV was chosen for EELS mapping of Li_x_C_6_. **d**–**f** The strain mapping and **g**–**i** EELS mapping of Li_x_C_6_ in the graphite discharged at 1 C and 6 C, and charged after the 70th cycle at 6 C, respectively

Discharging at higher rates, such as 1 C and 6 C, leads to a further increase in the polarization and reduces the intercalation capacity (Fig. 3a). The strain mapping in Fig. 3d–e demonstrates that local strain is distributed at the subsurface of graphite, suggestive of shallow Li^+^ intercalation. Especially for the 6 C-discharged graphite, the Li^+^ intercalation depth is within 5 nm of the subsurface, yielding an ultra-low reversible capacity of 18.6 mAh g^−1^ (Fig. 3b, e). EELS mapping was also applied to distinguish the Li^+^ distribution in the lithiated graphite based on the characteristic Li spectrum of Li_x_C_6_ (Fig. 3c), which shows consistent results with the strain mapping that Li^+^ is unevenly distributed and aggregated in the subsurface, especially at the fast-lithiated graphite (Fig. 3g–i). In contrast, Li^+^ is uniformly distributed in the slowly lithiated graphite at 0.05 C (Fig. S3). It is interesting that the reversible capacity increases gradually as a function of cycling number at 6 C and reaches 115.3 mAh g^−1^ at the 70th cycle, implying the slight enhancement of reaction kinetics. This is probably due to the gradual accumulation of residual defective structures (Fig. 3f) and formation of stable SEI layer (Fig. S4 and Table S1), which facilitates the Li^+^ diffusion through the particle bulk and interface.

These suggest that the fast lithiation is blocked by the sluggish reaction kinetics and stopped at the subsurface of graphite, resulting in uneven Li^+^ distribution and low reversible capacity. Engineering the bulk structure of graphite by creating more defective domains in situ (after multicycles) or artificially [22, 24–27] can slightly enhance the reaction kinetics but is not effective. This indicates that Li^+^ diffusion into the particle bulk might not be the main rate-determining step for the fast charging of graphite in this case.

### Particle Diffusion

To quantify the rate of Li^+^ diffusion through the graphite particle, PRT was applied to measure the chemical diffusion coefficient of the Li^+^ in graphite at different stages. Compared with other electrochemical techniques, PRT is more convenient and accurate because single variant that is the open-circuit voltage is used without any other estimations, such as the real surface area of the electrode and the molar volume of the lithiated material as requested for galvanostatic intermittent titration technique (GITT) [19]. In addition, the current during the OCV test is negligible and has little influence on the electrochemical polarization and reactions.

**Fig. 4:**
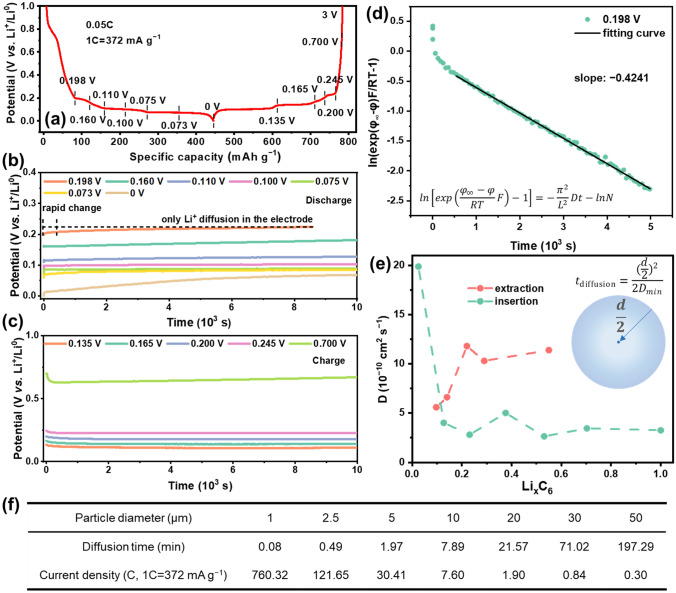
**a** The potential profile of graphite lithiated at 0.05 C. **b**–**c** Potential relaxation profiles of graphite at different (**b**) discharge /(**c**) charge states in (**a**). **d** Typical curve of $\ln \left[ {\exp \left( {\frac{{\varphi_{\infty } - \varphi }}{RT}F} \right) - 1} \right]$ *vs.* Time of graphite discharged to 0.198 V in (**b**). The solid line indicates the linear fitting result. **e** Calculated Li^+^ chemical diffusion coefficient *D* in the lithiated graphite (Li_x_C_6_, 0 ≤ x ≤ 1) during the insertion and extraction process. ** f** Li^+^ diffusion time and corresponding current density calculated based on the minimum Li^+^ chemical diffusion coefficient (*D*_*min*_ = 2.64 × 10^−10^ cm^2^ s^−1^)

Figure 4 shows the potential relaxation profile and the calculated Li^+^ chemical diffusion coefficients at different discharge/charge states. After removing the current, the voltage increases/decreases gradually and then becomes stable (Fig. 4b–c). The rapid voltage change is attributed to the Li^+^ diffusion in the electrolyte and the short-range interface, while the subsequent slow change is ascribed to the Li^+^ diffusion through the long-range particle and electrode; the former is dominant for a thin and porous electrode. Therefore, the chemical diffusion coefficient *D* of the Li^+^ in graphite can be calculated from the linear slope of $\ln \left[ {\exp \left( {\frac{{\varphi_{\infty } - \varphi }}{RT}F} \right) - 1} \right]$ over time [19] (Fig. 4d). Figure 4e and Table S2 show that, with the continuous Li^+^ intercalation, the diffusion coefficient firstly drops from initial 19.87 × 10^−10^ to 2.64 × 10^−10^ cm^2^ s^−1^, then slightly increases to 3.45 × 10^−10^ cm^2^ s^−1^, and finally becomes stable at 3.25 × 10^−10^ cm^2^ s^−1^. The slight rise of Li^+^ diffusion coefficient is probably owing to the increased defective structures and strains in the graphite facilitating the Li^+^ diffusion. Based on the lowest Li^+^ diffusion coefficient (2.64 × 10^−10^ cm^2^ s^−1^), the time for Li^+^ diffusing from the surface to the bulk center of graphite with a diameter of 10 μm is estimated to be about 7.89 min (~ 7.60 C, Fig. 4f), less than the requested time at 6 C (10 min). This indicates that the Li^+^ diffusion through the graphite bulk is quick enough to support the fast intercalation at least 6 C even though the above diffusion time may be underestimated due to the simple model applied. Compared with the intercalation process, Li^+^ has higher diffusion coefficients during the deintercalation, demonstrating that the Li^+^ extraction is much easier than the Li^+^ insertion (Fig. 4e and Table S3). Therefore, the low capacity and large polarization of graphite anode during fast Li^+^ intercalation are mainly due to the sluggish Li^+^ diffusion across the interface rather than in the bulk.

### Interfacial Diffusion

**Fig. 5:**
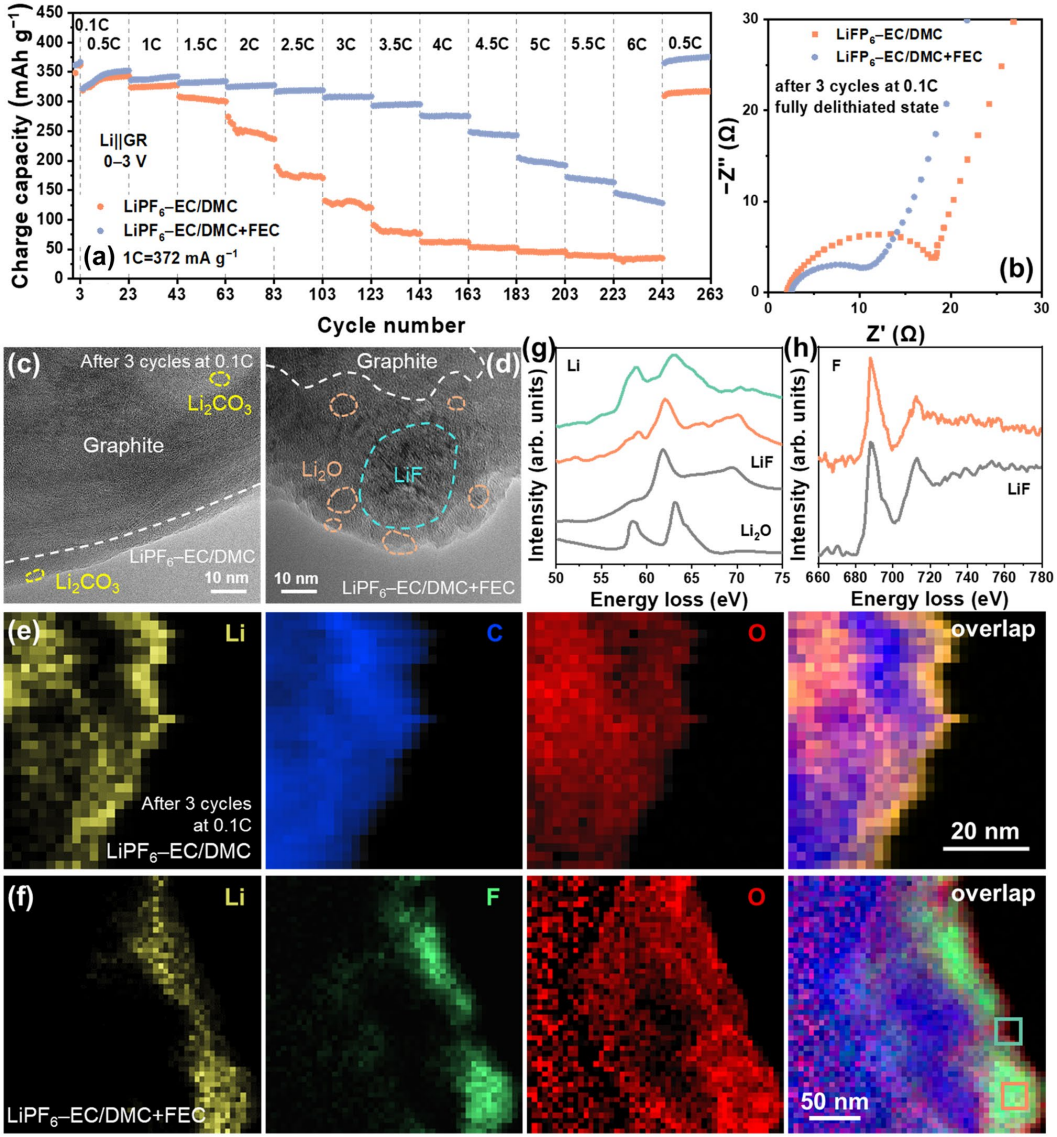
**a** The rate performances of Li||graphite cells. **b** EIS of graphite electrodes (fully delithiated state) tested by three-electrode cell after three cycles at 0.1 C. **c-d** HRTEM images and **e**–**f** EELS mappings of SEI on graphite after three cycles at 0.1 C with LiPF_6_–EC/DMC and LiPF_6_–EC/DMC + FEC electrolytes, respectively. **g**–**h** The corresponding **g** Li K-edge and **h** F K-edge EELS spectra from the regions in f labeled by green and orange boxes. The reference spectra of Li_2_O and LiF (gray lines in **g** and **h**) are obtained from references [36, 37]

The interfacial Li^+^ diffusion referred here includes the desolvation of the solvated Li ions and their passing through across the SEI layer given that these two processes are likely to occur concurrently and are difficult to separate from each other due to the porous nature of SEI layer [9, 12–14]. Since both of them are highly dependent on the electrolyte chemistry [18, 28–31], fluoroethylene carbonate (FEC) was used as an electrolyte additive (LiPF_6_–EC/DMC + 10 vol% FEC) to tune the electrolyte composition forming a LiF-rich SEI layer, which has been proven conducive to accelerating the Li^+^ diffusion kinetics in the SEI layer [32–34]. As expected, FEC addition drastically increases the reversible capacity at high rates, especially above 2 C (Figs. 5a and S5), and reduces the interfacial resistance (Fig. 5b). The distribution of relaxation times (DRT) analysis was conducted to decouple the individual contribution of *R*_SEI_ and *R*_ct_ from the overlapped spectra (Fig. 5b) by time characteristics (Fig. S6). Compared with the slightly decrease of *R*_ct_ (4.77 *vs.* 4.44 Ω) in FEC-containing electrolyte, the large discrepancy in *R*_SEI_ (13.77 *vs.* 4.67 Ω) suggests that the addition of FEC mainly accelerates the ion transport kinetics across the SEI layer (Table S4). In Li||graphite half cells (abbreviated as Li||GR), graphite with FEC-containing electrolyte delivers a capacity of 276.2 mAh g^−1^, while a capacity of 62.0 mAh g^−1^ is obtained in the conventional electrolyte at 4 C. Even at 6 C, 145.5 mAh g^−1^ is achieved in the FEC-containing electrolyte (Fig. 5a). To eliminate the effect lithium metal [35], the electrochemical performance of graphite||LiFePO_4_ full cells (abbreviated as GR||LFP, and the areal capacity ratio of positive to negative electrodes (P/N ratio) is 1.2) was tested at a charge/discharge voltage range of 0–3.45 V to avoid lithium metal deposition (Fig. S5). When the current density exceeds 1.5 mA cm^−2^, the reversible capacity of GR||LFP using LiPF_6_–EC/DMC electrolyte decreases rapidly, which is only 59.3 mAh g^−1^ at 2 mA cm^−2^, while it retains a reversible capacity of 300.7 and 70.5 mAh g^−1^ at 2.0 and 6.0 mA cm^−2^ in LiPF_6_–EC/DMC + FEC electrolyte, respectively. This large enhancement further demonstrates that the interfacial Li^+^ diffusion dominates the rate performance of graphite.

Compared with the graphite cycled in LiPF_6_–EC/DMC electrolyte, the graphite surface is coated with 50–100 nm nanoparticles and 400–500 nm nanospheres in FEC-containing electrolyte (Fig. S7). The composition and structure of the SEI layers in these two electrolytes were revealed by cryo-TEM and XPS (Figs. 5 and S8–S9). More LiF was found in the F 1*s* spectrum of FEC-containing electrolyte due to the decomposition of FEC (Fig. S8). The energy-dispersive spectroscopy (EDS) indicates that the SEI formed in the FEC-containing electrolyte contains a higher content of F (24.7% *vs.* 3.7%, Fig. S9) concentrated in particles dispersed on the graphite surface (Figs. S7 and S9). In contrast, less F is detected and uniformly distributed on the graphite cycled in the conventional electrolyte (Fig. S9).

High-resolution TEM (HRTEM) image (Fig. 5c) reveals that the SEI layer formed in the FEC-free electrolyte is thin (3–7 nm), consisting of amorphous organic species and some Li_2_CO_3_ nanograins, as is verified with the EELS mapping (Fig. 5e). For the SEI formed in FEC-containing electrolyte, crystalline LiF nanoparticles are present with a diameter of around 20 nm, which are surrounded by crystalline Li_2_O nanograins and amorphous organic species (Fig. 5d). Such core–shell structure is visualized by the EELS mapping (Fig. 5f), where F is concentrated in the center, while O is rich on its surface. The internal LiF phase and external Li_2_O phase were further evidenced with their characteristic EELS spectra (Fig. 5g–h). This LiF-rich SEI layer is 44–56 nm thick and dominantly located on the surface of the graphite (Fig. 5f), which regulates the interface properties and facilitates the Li^+^ diffusion across it.

### Electrode Diffusion

**Fig. 6:**
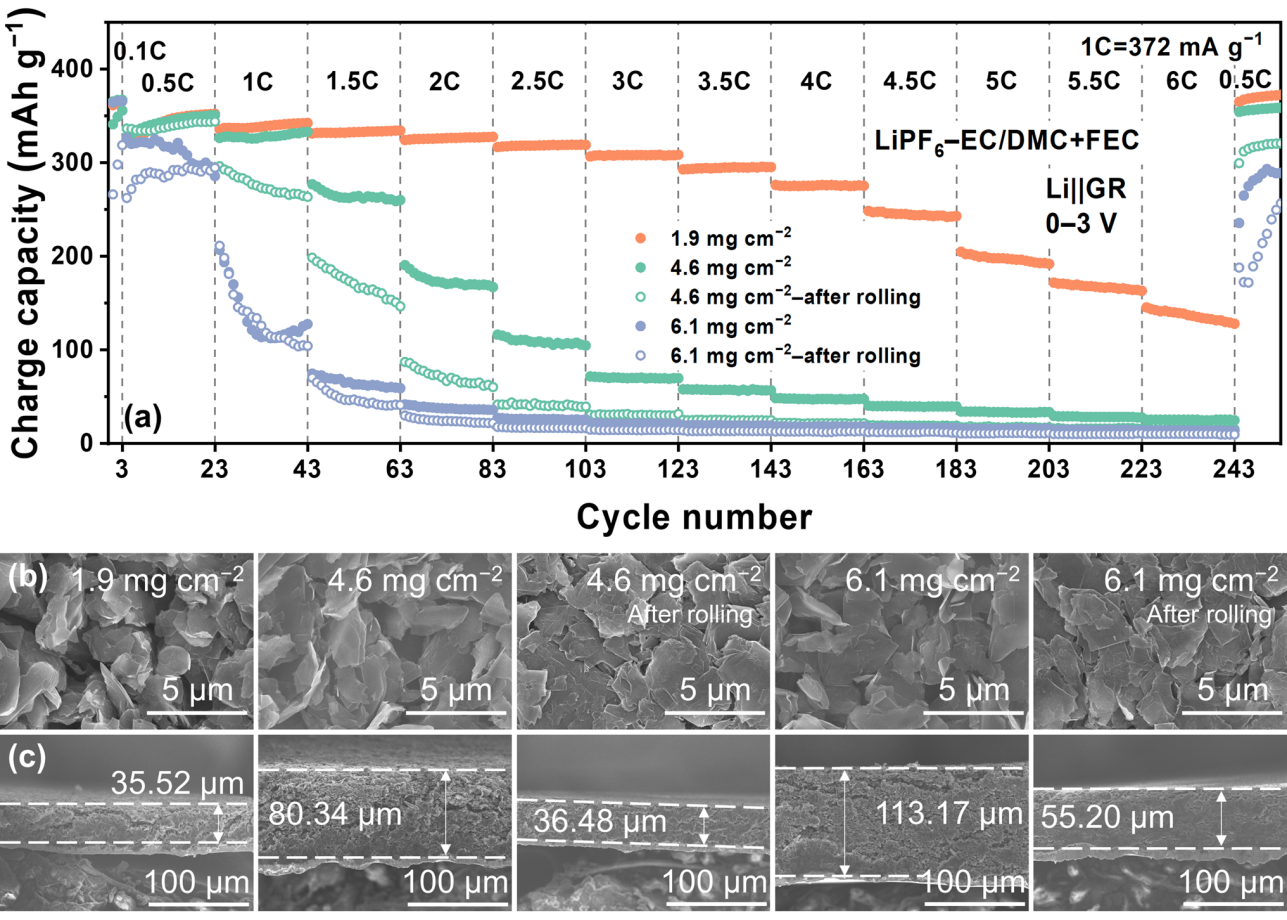
**a** The rate performances of Li||graphite cells with LiFP_6_–EC/DMC + FEC electrolytes using different graphite electrodes. **b**–**c** The corresponding surface morphology and cross-sectional images of different graphite electrodes

Note that the above analysis is based on the thin graphite electrode without rolling. When the loading of active materials, electrode thickness and compactness were increased, the kinetics of Li^+^ diffusion in electrode becomes nonnegligible and even dominates the rate performance of graphite (Fig. 6a). Increasing the loading from 1.9 to 4.6 and 6.1 mg cm^−2^ dramatically decreases the reversible capacity at the high rates since the vertical Li^+^ diffusion length is elongated from 35.52 to 80.34 and 113.17 μm, respectively (Fig. 6b–c). After rolling, although the electrode thickness is shrunken to less than the half (Fig. 6b), the electrode compactness is largely enhanced (Fig. 6b), resulting in further capacity drop (Fig. 6a). When the loading of active materials is increased, the Li^+^ diffusion length will be increased accordingly and it takes longer time to reach the equilibrium potential in the thick electrode (Fig. S10), corresponding to 2.2 × 10^4^, 2.5 × 10^4^, and 3.2 × 10^4^ s for 1.9, 4.6, and 6.1 mg cm^−2^. These results indicate that for practical high-loading and compact graphite electrode, electrode engineering is essential to facilitate Li^+^ diffusion in electrode, such as vertically aligned graphite electrode [38, 39] and laser patterning [40]. Compared with conventional electrolyte, the thick graphite electrode with FEC-containing electrolyte exhibits much better rate performance (Fig. S11), suggesting that electrolyte regulation or interphase design is also necessary to boost fast Li^+^ intercalation into graphite.

### Discussion

Fast Li^+^ intercalation into graphite is limited by the Li^+^ diffusion through the graphite particle, interface, and electrode. Based on the experimentally measured Li^+^ diffusion coefficient (> 2.64 × 10^−10^ cm^2^ s^−1^) in the particle, 10-μm graphite is able to be fast charged at 6 C, while it reduces to 2 C for 20 μm graphite (Figs. 4f and S12). When 6-μm graphite flakes were used, for the thin graphite electrode (< 2 mg cm^−2^), the rate-determining step is governed by the interfacial Li^+^ diffusion, consisting of the desolvation and crossing through the SEI layer, which is highly dependent on the electrolyte chemistry. Construction of a fluorinated SEI layer is proven conducive to accelerating the interfacial Li^+^ diffusion and drastically enhancing the fast-charging performance of graphite. For thick and compact graphite electrode, Li^+^ diffusion in the electrode becomes the main reaction-determining steps. Therefore, to achieve high-performance graphite anode for fast-charging LIBs, the particle size of graphite, electrolyte, interface, and electrode configuration should be fully considered and well designed and matched.

## Conclusions

In combination of the cryo-TEM and other methods, we revealed the evolution of the bulk and interface structure of graphite upon fast Li^+^ intercalation and its correlation with the reaction kinetics and electrochemical performance. The fast-lithiated graphite structure consists of various staging structures, exhibiting a significant heterogeneity in the macroscopic and microscopic scales. The Li^+^ particle diffusion coefficient is estimated large enough to enable the fast charging at a rate of 6 C if the particle size is less than 10 μm. Therefore, the Li^+^ interface diffusion dominates the reaction kinetics at high rates in thin graphite electrode, which is highly dependent on the electrolyte chemistry and can be enhanced with the fluorinated SEI layer. However, Li^+^ diffusion through the electrode cannot to be neglected for thick graphite electrode. Therefore, the combination of the electrode engineering and electrolyte modification can promote the diffusion of Li^+^ in graphite electrode and interface. These findings renew the insights into the micro- and macrostructure of the fast-lithiated graphite, decipher the bottleneck for the sluggish reaction kinetics, and provide explicit guidance to enhance the fast-charging performance of graphite anode.

### Supplementary Information

Below is the link to the electronic supplementary material.

Supplementary file1 (PDF 1475 kb)
