# Supplementary material for: Kinetic Limits of Graphite Anode for Fast-Charging Lithium-Ion Batteries
Source: Nanomicro Lett. 2023 Sep 22;15:215. doi: 10.1007/s40820-023-01183-6 (PMC10516836; doi:10.1007/s40820-023-01183-6)
Supplement: Supplementary file 1 — Supplementary file1 (PDF 1475 kb) [file 40820_2023_1183_MOESM1_ESM.pdf]

Supporting Information for

Kinetic Limits of Graphite Anode for Fast-Charging Lithium-Ion Batteries

Suting Weng[1, 2], Gaojing Yang[1, 2], Simeng Zhang[1, 3], Xiaozhi Liu[1], Xiao Zhang[1, 3], Zepeng Liu[1, 2], Mengyan Cao[1, 3], Mehmet Nurullah Ateş[4], Yejing Li[1, *], Liquan Chen[1], Zhaoxiang Wang[1, 2, 3, *] and Xuefeng Wang[1, 2, 3, 5, *]

[1] Beijing National Laboratory for Condensed Matter Physics, Institute of Physics, Chinese Academy of Sciences, Beijing, 100190, P. R. China

[2] School of Physical Sciences, University of Chinese Academy of Sciences, Beijing, 100049, P. R. China

[3] College of Materials Science and Opto-Electronic Technology, University of Chinese Academy of Sciences, Beijing, 100049, P. R. China

[4] Department of Chemistry, Boğazici University, Bebek, Istanbul, 34342, Türkiye

[5] Tianmu Lake Institute of Advanced Energy Storage Technologies Co. Ltd., Liyang, 213300, P. R. China

*Corresponding authors. E-mail: wxf@iphy.ac.cn (X. Wang); zxwang@iphy.ac.cn (Z. Wang); liyejing26@gmail.com (Y. Li)

Supplementary Figures and Tables

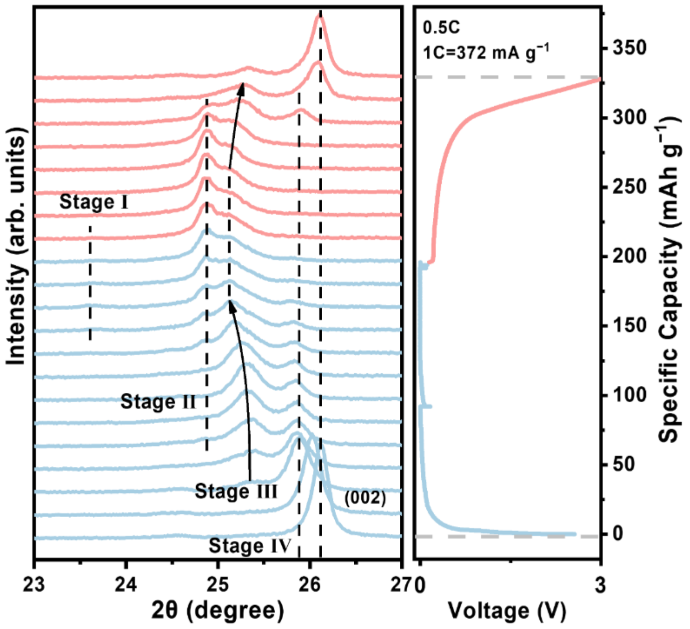

Fig. S1 The structural evolution of the graphite during electrochemical lithiation and delithiation at 0.5C

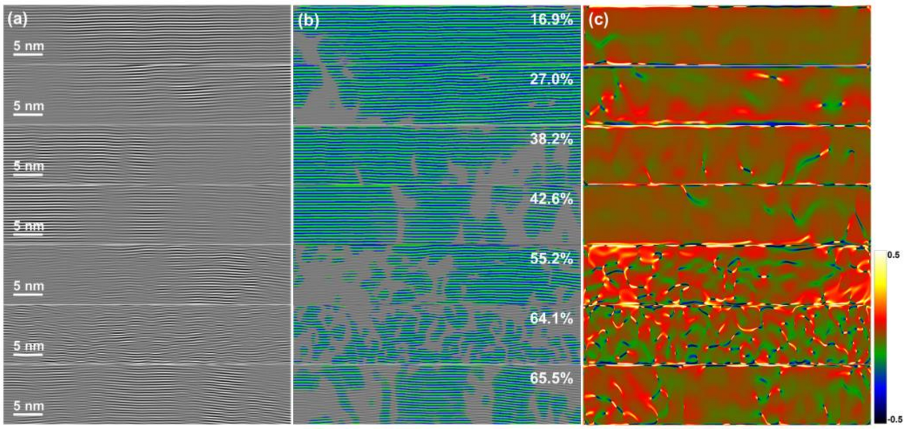

Fig. S2 **a** Inverse fast Fourier transform images, the corresponding **b** area of the defective domains and **c** strain mapping of different graphite regions lithiated at 0.5C. The color from red-to-yellow-to-white in the strain mapping represents the gradually increased tensive strain while that from green-to-blue-to-black indicates the gradually enhanced compressive strain

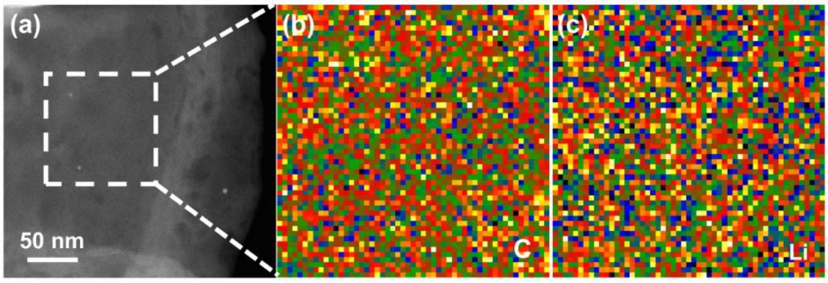

Fig. S3 **a** STEM-ADF image. **b-c** The corresponding EELS mapping of C and Li K-edge of graphite lithiated at 0.05C

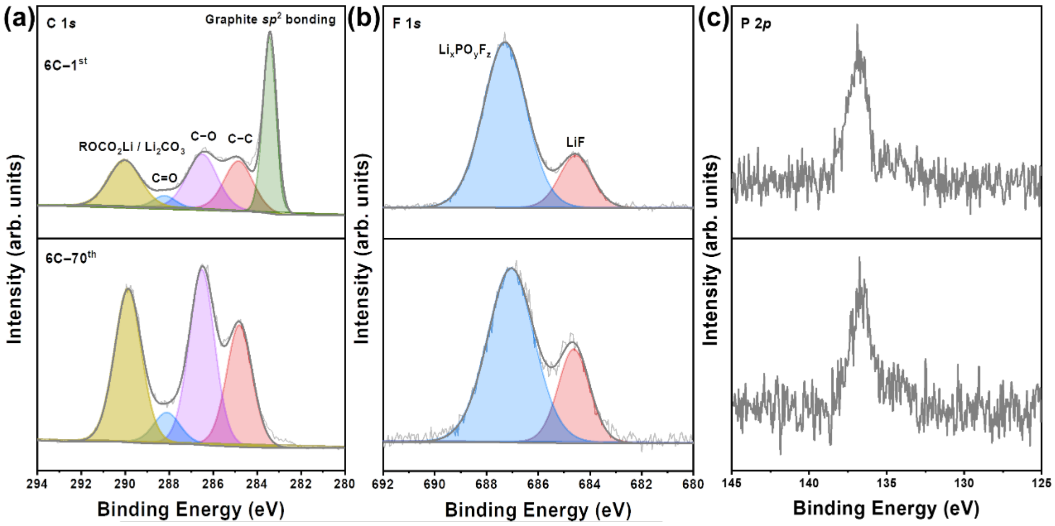

Fig. S4 XPS spectra of the SEI layers formed on graphite after 1st and 70th cycle at 6C. **a** C 1*s*, **b** F 1*s*, and **c** P 2*p*. The results show that the SEI components change slightly (Table S1) while it becomes thicker since the *sp*^{2} bonding signal around 283.5 eV from

the graphite bulk is absent after 70 cycles (Fig. S4a)

Table S1 The relative content of specific species in the SEI layer based on the XPS spectra in Fig. S4

Species	6C-1st	6C-70th
C−C	29.50%	24.77%
C−O	34.49%	35.68%
C=O	5.14%	6.36%
$ROCO_2Li/Li_2CO_3$	30.87%	33.19%
LiF	19.04%	26.24%
$Li_xPO_yF_z$	80.96%	73.76%

Table S2 Lithium content and its corresponding chemical diffusion coefficient of graphite at different discharge potentials

Potential (V *vs.* Li^+/Li^0)	0.198	0.160	0.110	0.100	0.075	0.073	0
x in Li_xC_6	0.025	0.127	0.231	0.376	0.531	0.703	1
D (10^{-10} $cm^2\ s^{-1}$)	19.87	4.02	2.81	5.01	2.64	3.45	3.25

Table S3 Lithium content and its corresponding chemical diffusion coefficient of graphite at different charge potentials

Potential (V *vs.* Li^+/Li^0)	0.700	0.245	0.200	0.165	0.135
x in Li_xC_6	0.096	0.139	0.217	0.288	0.551
D (10^{-10} $cm^2\ s^{-1}$)	5.59	6.61	11.80	10.30	11.39

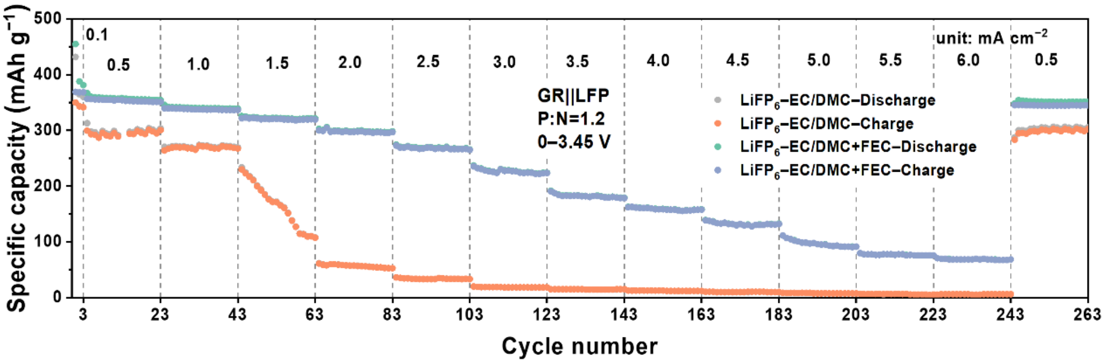

Fig. S5 The rate performances of graphite||$LiFePO_4$ cells

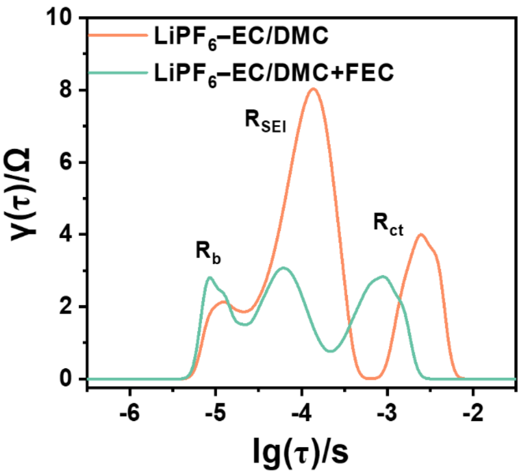

Fig. S6 The corresponding distribution of relaxation times (DRT) analysis of the electrochemical impedance spectra in Fig. 5b

Table S4 The corresponding R_b, R_{SEI}, and R_{ct} values obtained by integrating the natural logarithm ln(τ) of the time constant τ with γ(τ)

Resistance (Ω)	$LiPF_6$–EC/DMC	$LiPF_6$–EC/DMC+FEC
R_b	2.35	2.79
R_{SEI}	13.77	4.67
R_{ct}	4.77	4.44

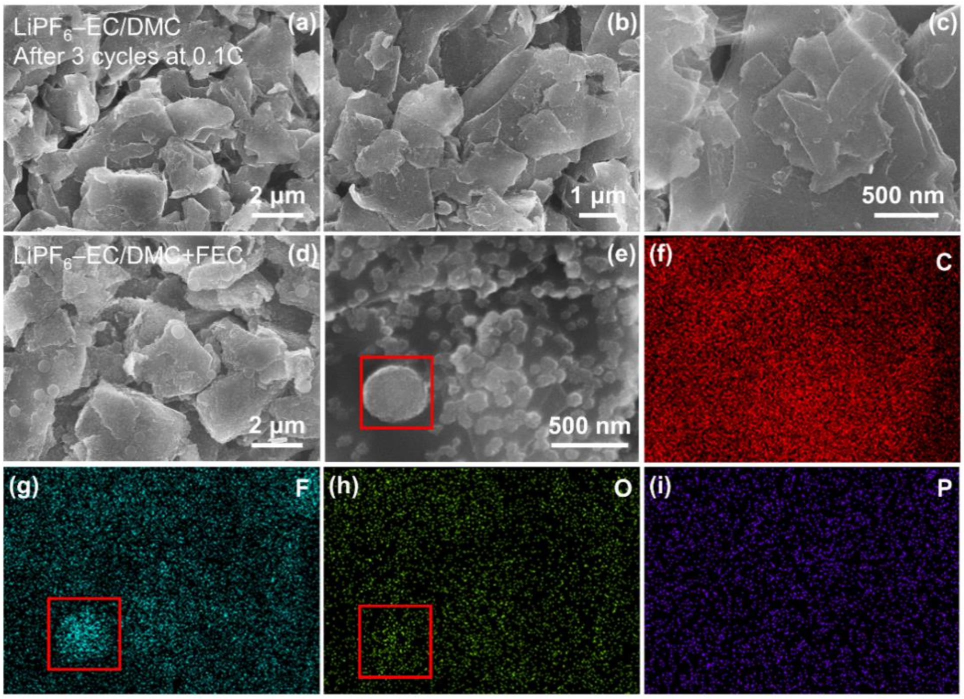

Fig. S7 SEM images with different magnifications of graphite cycled in **a-c** $LiFP_6$–EC/DMC and **d-e** $LiFP_6$–EC/DMC+FEC electrolytes after 3 cycles at 0.1C. **f-i** The corresponding elements distribution of **e**

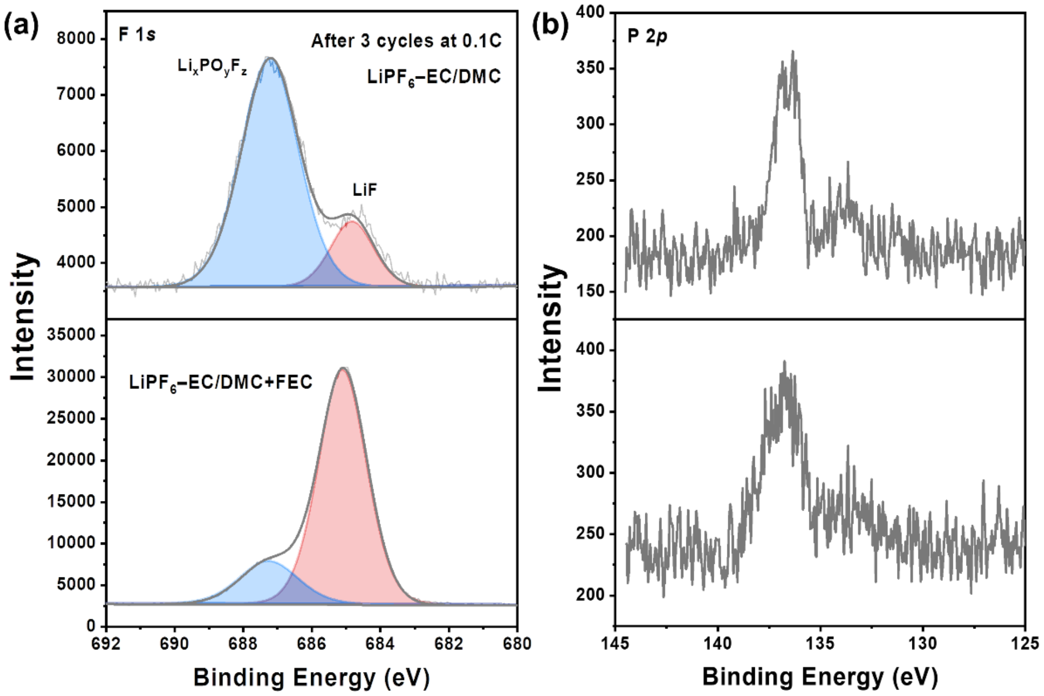

Fig. S8 XPS spectra of the SEI layers formed on graphite in $LiFP_6$–EC/DMC and $LiFP_6$–EC/DMC+FEC electrolytes after 3 cycles at 0.1C. **a** F 1*s* and **b** P 2*p*

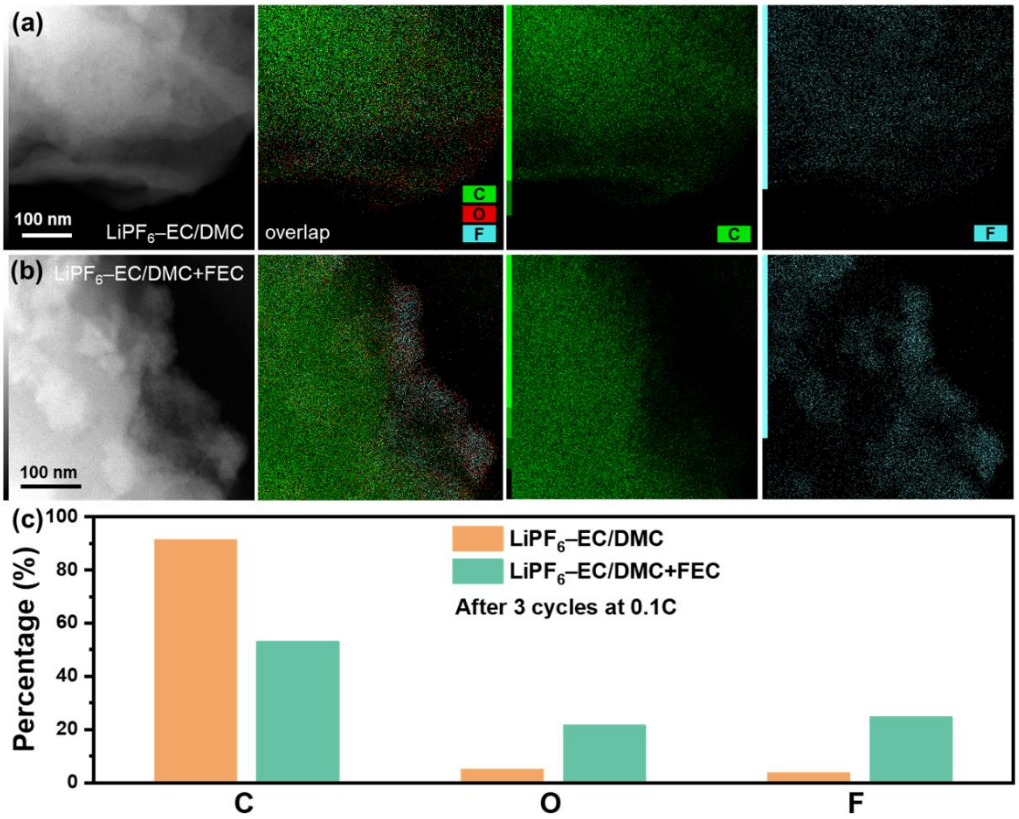

Fig. S9 EDS mapping of SEI layers formed on graphite in **a** $LiFP_6$–EC/DMC and **b** $LiFP_6$–EC/DMC+FEC electrolytes after 3 cycles at 0.1C. **c** Corresponding different elemental percentage of SEI layers

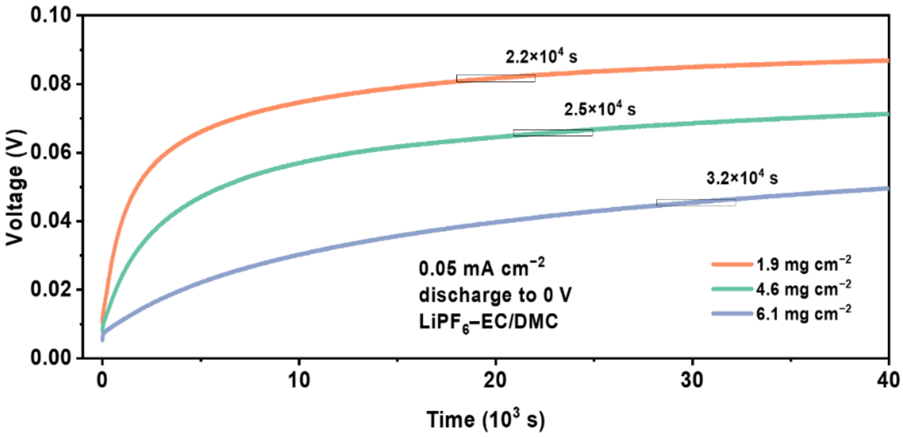

Fig. S10 Potential relaxation profiles of graphite electrodes with different thicknesses discharged to 0 V at the same areal current density

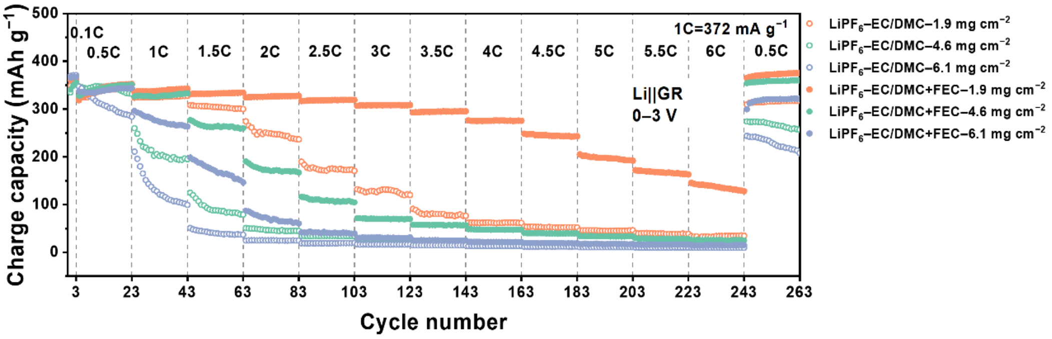

Fig. S11 The rate performances of different thickness graphite electrodes in Li||graphite cells using $LiFP_6$–EC/DMC and $LiFP_6$–EC/DMC+FEC electrolytes

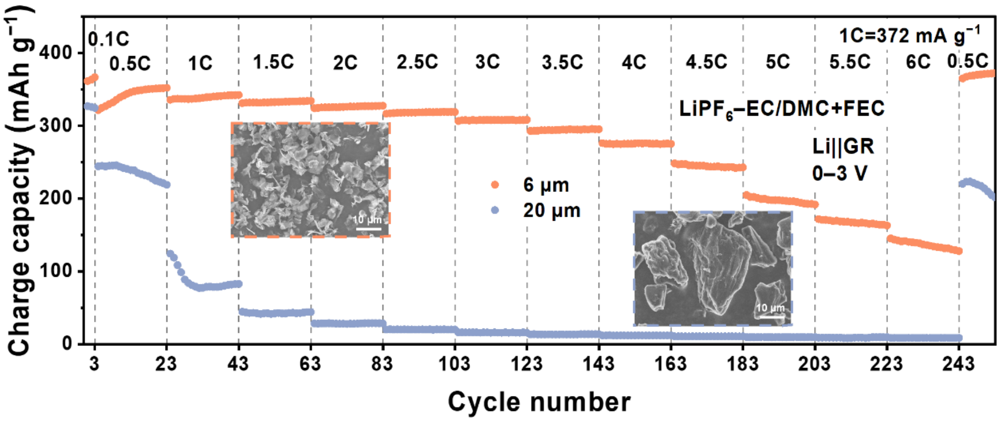

Fig. S12 The rate performances of Li||graphite cells with $LiFP_6$–EC/DMC+FEC electrolytes using different particle sizes of graphite
